# N6-Methyladenosine Modifications in the Female Reproductive System: Roles in Gonad Development and Diseases

**DOI:** 10.7150/ijbs.66218

**Published:** 2022-01-01

**Authors:** Hongbei Mu, Huiying Li, Yu Liu, Xiaofei Wang, Qiaojuan Mei, Wenpei Xiang

**Affiliations:** 1 Institute of Reproductive Health, Tongji Medical College, Huazhong University of Science and Technology, Wuhan, China.; 2 Center of Reproductive Medicine, Tongji Medical College, Huazhong University of Science and Technology, Wuhan, China.

**Keywords:** N6-methyladenosine, epigenetics, oogenesis, fertility, Female reproductive system

## Abstract

N6-methyladenosine (m^6^A) is the most prevalent chemical modification in eukaryotic messenger RNAs. By participating in various RNA-related bioprocesses including RNA decay, splicing, transport and translation, m^6^A serves as a pivotal regulator of RNA fate and plays an irreplaceable role in cellular activities. The m^6^A modifications of transcripts are coordinately regulated by methyltransferase “writers” and demethylase “erasers”, and produce variable effects via different m^6^A reading protein “readers”. There is emerging evidence that m^6^A modifications play a critical role in a variety of physiological and pathological processes in the female reproductive system, subsequently affecting female fertility. Here, we introduce recent advances in research on m^6^A regulators and their functions, then highlight the role of m^6^A in gonad development and female reproductive diseases, as well as the underlying mechanisms driving these processes.

## Introduction

More than 170 kinds of RNA modifications were found to be widely distributed among various organisms 1, which are collectively known as the 'epitranscriptome'. Substantial studies aimed to uncover the mechanisms and characterizations of these modifications, such as N6-methyladenosine (m^6^A), N1-methyladenosine, 5-methylcytidine and pseudouridine 2, unveiling a brand-new landscape of epigenetic regulatory mechanisms.

Among all of these modifications, m^6^A, which has been reported as early as 1974 3, 4, was considered to be the most prevalent chemical modification in eukaryotic messenger RNAs 2, 5, 6. The m^6^A modification is preferentially enriched around stop codons, 5'- and 3'-untranslated regions (UTR), as well as in long internal exons with the consensus motif RRACH (R = A or G, H = A, C or U) 7, 8. Due to its involvement in various RNA-related bioprocesses, including RNA decay, splicing, transport and translation 9-12, m^6^A is a pivotal regulator of RNA fate and plays an irreplaceable role in cell differentiation, development, metabolism, stress response and other cellular activities 2, 13-15.

Epigenetic molecules and signaling pathways in the female reproductive system have various effects and are important for female fertility 16-19. With the deepening of m^6^A research, there is emerging evidence for the involvement of m^6^A in various physiological and pathological processes in the female reproductive system, including oogenesis and diseases, subsequently affecting female fertility. Here, we introduce recent advances in research on m^6^A regulators and their functions, and highlight the role of m^6^A in oogenesis and female reproductive diseases, as well as the underlying mechanisms driving these processes.

## Regulators of m^6^A

**Figure 1:**
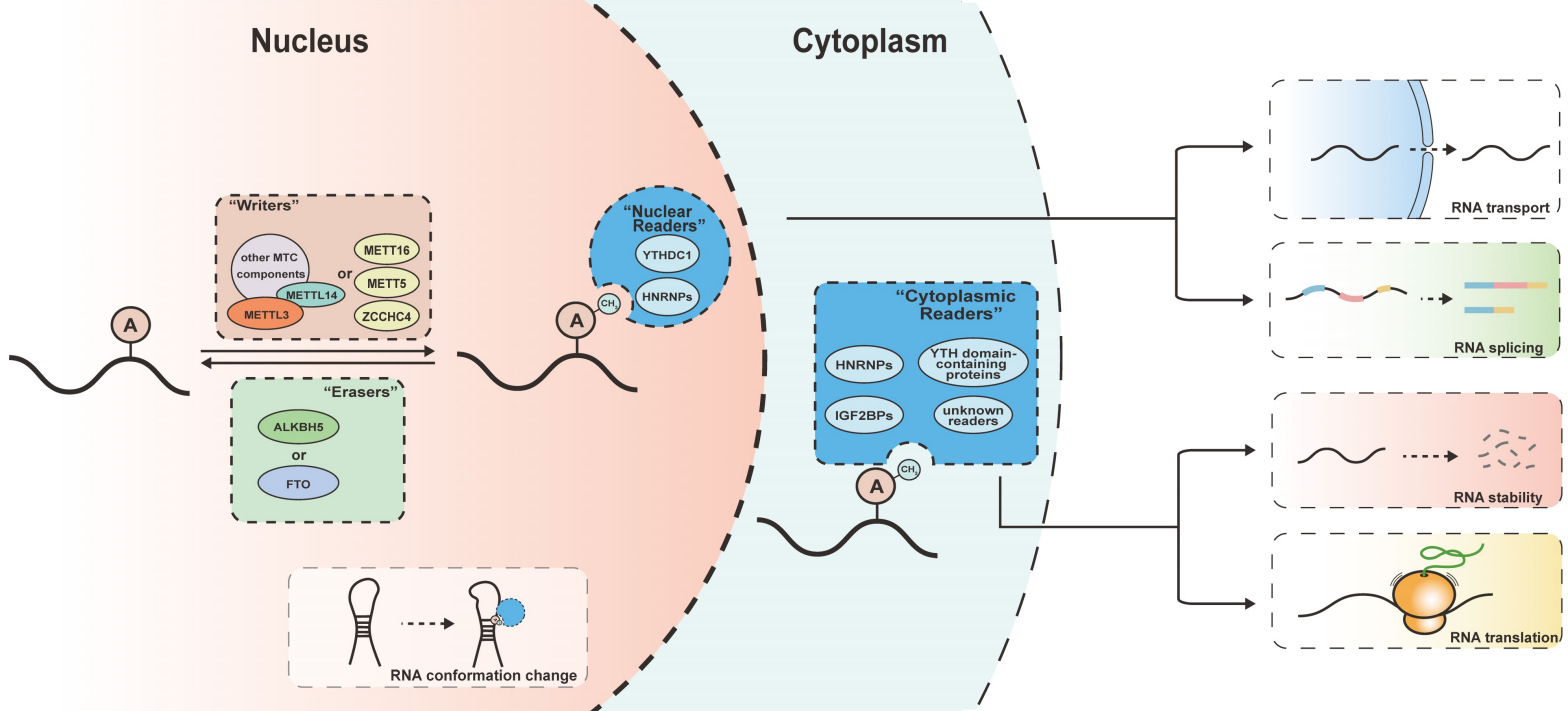
** mechanism of m^6^A modifications.** The m^6^A modification is catalyzed by writers including multicomponent methyltransferase complex (METTL3, METTL14 and other auxiliary components) and several independent methyltransferase (METTL16, METTL5, ZCCHC4), removed by erasers (ALKBH5 or FTO) and recognized by m^6^A readers (YTH domain-containing proteins, HNRNPs and IGF2BPs). Readers bind to their target RNAs and meditate various downstream effect including RNA transport, splicing, degradation and translation. The m^6^A modifications can regulate the interaction between RNA and m^6^A readers by meditating the conformation changes of RNAs.

The m^6^A modification is a reversable process dynamically controlled by m^6^A writers and erasers, and its variable downstream effects rely on the recognition of m^6^A modifications by m^6^A readers **(Figure 1)**.

### m^6^A writers

The introduction of the m^6^A modification is accomplished by the multicomponent methyltransferase complex (MTC). The MTC consists of the catalytic subunit METLL3 and auxiliary subunits including METTL14, WTAP, KIAA1429, RBM15 and ZC3H13 20. METTL3 serves as the core component of the MTC by installing the m^6^A modification by adding a methyl group to the N6 position of adenine. METTL14 forms a stable heterodimer core complex with METTL3, enabling the latter to recognize its substrates 21-23. WTAP is indispensable for the correct localization of the METTL3-METTL14 complex in the nuclear speckles, which guarantees the correct continuation of m^6^A-related processes 24, 25. KIAA1429 and RBM15 maintain the m^6^A levels by recruiting the MTC to specific RNA regions 24, 26, 27. ZC3H13 and its *Drosophila* homolog Flacc function as a bridge between the MTC and its mRNA targets, and guide RMB15 to WTAP to facilitate m^6^A modification 28, 29. Hakai is a newly identified member of the MTC in *Drosophila* and human cells that helps stabilize other subunits of the MTC through its ubiquitination domain 30. Given that METTL3 only produces a part of RNA m^6^A modifications, there must be other writers meditating the other methyl transfer reactions 31. METTL16 is a newly identified m^6^A methyltransferase targeting pre-mRNAs and various non-coding RNAs 32, 33. It consists of two structural domains, whereby the N-terminal methyltransferase domain independently recognizes the 5′-UACAGAGAA motif and the C-terminal vertebrate conserved region binds to internal stem-loops that facilitate the methyltransferase activity of METTL16^34^. In addition, METTL5 and ZCCHC4 were found to be m^6^A writers that respectively act on the 18S and 28S rRNAs in an MTC-independent manner. METTL5 forms a heterodimer with TRMT112 to maintain its metabolic stability and serves as an exclusive m^6^A rRNA methyltransferase.^35^

### m^6^A Erasers

Erasers are demethylases that remove m^6^A modifications in a ferrous iron and α-ketoglutaric acid dependent manner, dynamically regulating the m^6^A labeling of mRNAs and other types of transcripts in the nucleus. Erasers determine the m^6^A levels of transcripts together with m^6^A writers, thus modulating the subsequent effects of m^6^A readers. There is evidence that FTO controls RNA splicing by modulating he interaction of RNAs with SRSF2 protein 36. ALKBH5, another known m^6^A eraser, has been proved to colocalize with mRNA-processing factors in nuclear speckles, and it plays critical roles in RNA transport, synthesis and stability. ALKBH5-dependent demethylation precisely controls the splicing process and length of the 3'-UTR region 37.

### m^6^A Readers

Recognition and binding of m^6^A readers to their target RNAs is required for m^6^A modification to execute its important functions in regulating RNA fate. The m^6^A readers discovered to date include YT521-B homology (YTH) domain-containing proteins, heterogeneous nuclear ribonucleoproteins (HNRNPs), insulin-like growth factor 2 mRNA-binding proteins (IGF2BPs) and eukaryotic initiation factor 3 (eIF3)^38^, ^39^.

These m^6^A readers take part in different RNA metabolic processes and affect different aspects of RNA behavior. YTH domain-containing proteins, the most researched m^6^A readers, recognize m^6^A modifications through the YTH domain and subsequently participate in various RNA-related processes 40. YTHDF1 positively regulates the translation of its targeted RNAs in a m^6^A-denpend manner 11, 41, YTHDF2 selectively induces the degradation of m^6^A-modifed mRNAs, decreasing the abundance of its targets 12, 42, while YTHDF3 facilitated decay and translation of RNAs by interacting with YTHDF1 and YTHDF2 43, 44. As a nuclear m^6^A reader, YTHDC1 meditates nuclear RNA export and is required for alternative splicing 45, 46. YTHDC2 promotes translational activities and also accelerates the degradation of its targets, showing a double-sided effect 47. HNRNPs and IGF2BPs are also protein families that function as m^6^A readers. Specifically, the binding affinity of HNRNPC for transcripts can be increased by m^6^A-mediated RNA structure alteration, leading to the m^6^A-switch effect 48. HNRNPA2B1 binds RNA through its RNA recognition motif and recognizes m^6^A-modified RNA in a non-specific manner, enhancing METTL3-dependent microRNA processing and production 49, 50. HNRNPG interacts with the phosphorylated carboxy-terminal domain of RNA polymerase II through its RGG motifs and co-transcriptionally regulates alternative splicing 51. IGF2BPs are a group of RNA binding proteins that selectively bind m^6^A-modified RNA and share overlapping RNA targets. IGF2BPs enhance mRNA stability and promote mRNA translation, thus upregulating gene expression 52-54.

## Biological functions of m^6^A signals

The introduction of m^6^A into transcripts mainly occurs in the nucleus, making it a nuclear imprint of RNA fate 55, 56. The m^6^A levels determined by the m^6^A MTC and m^6^A erasers re closely related to RNA metabolism outcomes in the cytoplasm. Through reversible and dynamic methylation of adenine residues, m^6^A regulates the stability, splicing, transport and translation of RNAs 21, 22, playing an important role in a variety of physiological and pathological processes.

### m^6^A in mRNA stability

Possible involvement of m^6^A modification in regulating RNA stability was first explored in 1978. Researchers deduced that rapid elimination of m^6^A in the cytoplasm may be caused by the shorter half-life of mRNAs with a high m^6^A content 57. This hypothesis that mRNAs harboring more m^6^A were susceptible to degradation has been proved later, and *Mettl3* knockout, which significantly decreased the m^6^A levels of RNAs, remarkably increased their half-life 58.

There is substantial evidence that m^6^A readers are the main regulators of RNA stability. RNA decay takes place in cytoplasmic processing bodies (P bodies) 59, and YTHDF2 was found to colocalizes with P bodies and its cognate mRNAs were intended to have shorter half-live, demonstrating YTHDF2's role in destabilizing its mRNA targets. The deadenylation-dependent decay pathway was also a part of mechanism driving YTHDF2-mediated RNA decay 12, 42. YTHDF3 shares targets with its family members and promotes RNA degradation in cooperation with YTHDF2 44. Conversely, IGF2BPs enhances mRNA stability by recognizing m^6^A modifications, increasing the abundance of its mRNA targets. IGF2BPs silencing led to comprehensive downregulation of its target genes 54, 60.

It is worth mentioning that m^6^A modifications are also deposited in the RNAs of pseudogenes. These RNAs have higher m^6^A levels than their cognate mRNAs, which facilitates the degradation of processed pseudogene transcripts in the cytoplasm. Given that processed pseudogenes cannot be recognized and cleaned by canonical nonsense-mediated decay process, m^6^A-meditated cytosolic elimination of these nonfunctional RNAs provides a novel mechanism of RNA surveillance 61.

### m^6^A in mRNA splicing

The m^6^A modification plays a key role in sex determination in *Drosophila* by modulating the splicing of Sex lethal transcripts 62-64, revealing a novel posttranscriptional regulatory mechanism of gene expression. However, the effects and functions of m^6^A modification in RNA splicing in mammals have not been fully elucidated. Some researchers proposed that m^6^A does not have a major effect on splicing 58. However, there is accumulating evidence that m^6^A modification is involved in RNA splicing through multiple mechanisms. *Mettl3*-depletion partially affected RNA splicing in terms of frequency and type of alternative splicing events 15. The demethylases FTO and ALKBH5 control this process by regulating m^6^A levels in alternative exons cassettes and coding sequences, respectively^36^, ^65^. The nucleus-located m^6^A reader, YTHDC1, participates in the processing of transcripts together with splicing regulators such as CPSF6, SRSF3, and SRSF7 10, 66.

Mechanistically, the distribution pattern of m^6^A modifications may be a key factor driving the impact of m^6^A on alternative splicing. It was found that m^6^A is preferentially located in long exons, near the stop codons, and in 3′-UTR 48. Yet another study found that m^6^A was enriched near splice sites in both exonic and intronic regions 67. This differential distribution may lead to region-specific binding of splicing-related proteins, thereby mediating alternative splicing. The indirect effect of m^6^A on RNA splicing should also be taken into consideration. By controlling the expression of highly m^6^A-modified splicing regulators such as SON44, HNRNPC38 and HNRNPF 68, m^6^A may also alter RNA splicing outcomes without direct participation.

### m^6^A in mRNA transport

Correct subcellular localization of transcripts is closely related to their function, making m^6^A modification an important regulator of RNA fate by affecting nuclear RNA export. A conditional knockout of *Mettl14* led to nuclear retention of m^6^A-modified mRNAs by impairing the preferential binding of FMRP 69. *ALKBH5*-deficient cells with increased m^6^A levels showed accelerated nuclear export and massive cytoplasmic accumulation of mRNA, leading to significant changes in the ratio of nuclear and cytoplasmic mRNA content 9.The m^6^A reader YTHDC1 was found to enhance the binding of m^6^A-modified RNAs to nuclear export adaptor protein SRSF3 and mRNA export receptor NXF1, which facilitates the nuclear export of its target mRNAs^46^. These studies proved the involvement of m^6^A in nuclear RNA export, but its effect on the whole transcriptome needs to be further elucidated.

### m^6^A in mRNA Translation

Some researchers have shown that m^6^A facilitates mRNA translation by interacting with eukaryotic translation initiation factor eIF3 via two different pathways 11, 70. As one of the most important translation initiation factors in eukaryotic cells eIF3 is usually bound to the 5′ untranslated regions of mRNAs and recruits the small ribosomal subunit around its binding sites, thereby regulating translation initiation 71. It has been shown that the effect of the m^6^A reader YTHDF1 on protein expression is dependent on the eIF3-related translation machinery. YTHDF1-binding mRNAs showed a higher ribosome-bound fraction and translation efficiency 11. Conversely, *YTHDF1* silencing led to significantly downregulated protein production of its target mRNAs. Since YTHDF1 usually binds RNA close to stop codons in mammalian cells, the effect of 3'UTR-enriched YTHDF1 was based on eIF4G-induced spatial proximity to translation initiation sites 11. Direct eIF3 recruitment around 5'UTR also initiated mRNA translation. Cell-stress associated mRNAs contain m^6^A modifications around the 5'UTR region and exhibit enhanced cap-independent translation 70. Additionally, YTHDF1 was found to augment the translation of EIF3C in an m^6^A-dependent manner and form a YTHDF1-EIF3C axis promoting general protein synthesis 41.

Furthermore, METTL3, which was generally known as a methylase acting in the nucleus, also took part in mRNA regulation by interacting with the translation initiation machinery. Cytoplasmic METTL3 physically and functionally associates with eIF3 subunits, bridging its binding sites to the 5′-end of the mRNA to meditate mRNA circularization 72, 73. METTL3 depletion remarkably downregulated the expression of its target mRNAs 73.

### m^6^A in non-coding RNA

In addition to mRNAs, the deposition of m^6^A modifications also affects the fate of modified non-coding RNA (ncRNA) in various ncRNA-related bioprocesses 74. METTL3 induced m^6^A hyper-methylation facilitated stabilizing lncRNA, thus enhancing its expression 75, 76. FTO decreased the m^6^A level on the LINC00022 transcript and inhibited its degradation via the m^6^A reader YTHDF2^77^.HNRNPA2B1, a nuclear m^6^A reader, showed a METTL3-like effect in RNA splicing and promoted primary miRNA processing by interacting with the microRNA microprocessor complex protein DGCR8^50^. YTHDC1 was involved in the circRNA transport process. *YTHDC1* silencing led to significant nuclear retention of circNSUN2, which was rescued by enforced expression of wild-type YTHDC1 78. Moreover, the m^6^A modifications can initiate circRNA translation through YTHDF3-mediated recruitment of the translation initiation complex 79.

The m^6^A modification plays a key role in modulating interaction between ncRNAs and their targeted RNAs. For example, m^6^A modification promoted miR-133a binding to its targets and enhanced the miR-133a repression 80. LncRNA ILF3-AS1 recruited METTL3 to ILF3 mRNA, which increased its m^6^A level and strengthened its stability 81. The m^6^A-dependent RNA structural changes regulate the accessibility of RNA binding motifs of proteins to affect RNA-protein interactions, which is termed as “m^6^A-switch”. The m^6^A modification destabilizes hairpin stem structure of lncRNA MALAT1, thus making it more accessible for the RNA binding proteins 48.

## m^6^A in female gonad development and gametogenesis

Establishment of gonadal structure and function is dependent on the development of both germline cells and somatic cells. The oocyte and its surrounding somatic cells form the basic functional unit of the ovary, the follicle. Normal follicle development and gametogenesis are prerequisites for female fertility.

Follicle development, also known as folliculogenesis, is a long and complex process in which the recruited primordial follicle either develops into a mature Graafian follicle to ovulate or undergoes atresia. Folliculogenesis is synchronized with the growth and maturation of recruited oocytes, providing necessary sex steroids and establishing an appropriate microenvironment for oocytes.

In a broad sense, oogenesis is a highly sophisticated and coordinated process by which primordial germ cells (PGCs) develop into mature oocytes to prepare for fertilization. In humans, PGCs are specified as early as the peri-implantation window of the embryo at day 12 post-fertilization 82. PGCs exist transiently and soon differentiate into oogonia after undergoing genome-wide epigenetic reprogramming 83. The primary oocytes derived from diploid oogonia then enter meiosis and remain arrested in prophase I at birth. The prophase I oocytes have an intact germinal vesicle (GV) and remain dormant until gonadotropin stimulation in puberty, which can lead to germinal vesicle breakdown (GVBD). Following GVBD, the oocytes continue to complete meiosis I with formation of metaphase I spindles and separation of paired homologous chromosomes. Afterwards, the oocytes directly enter meiosis II and arrest for the second time in metaphase II (MII) until fertilization triggers the completion of meiosis.

**Figure 2:**
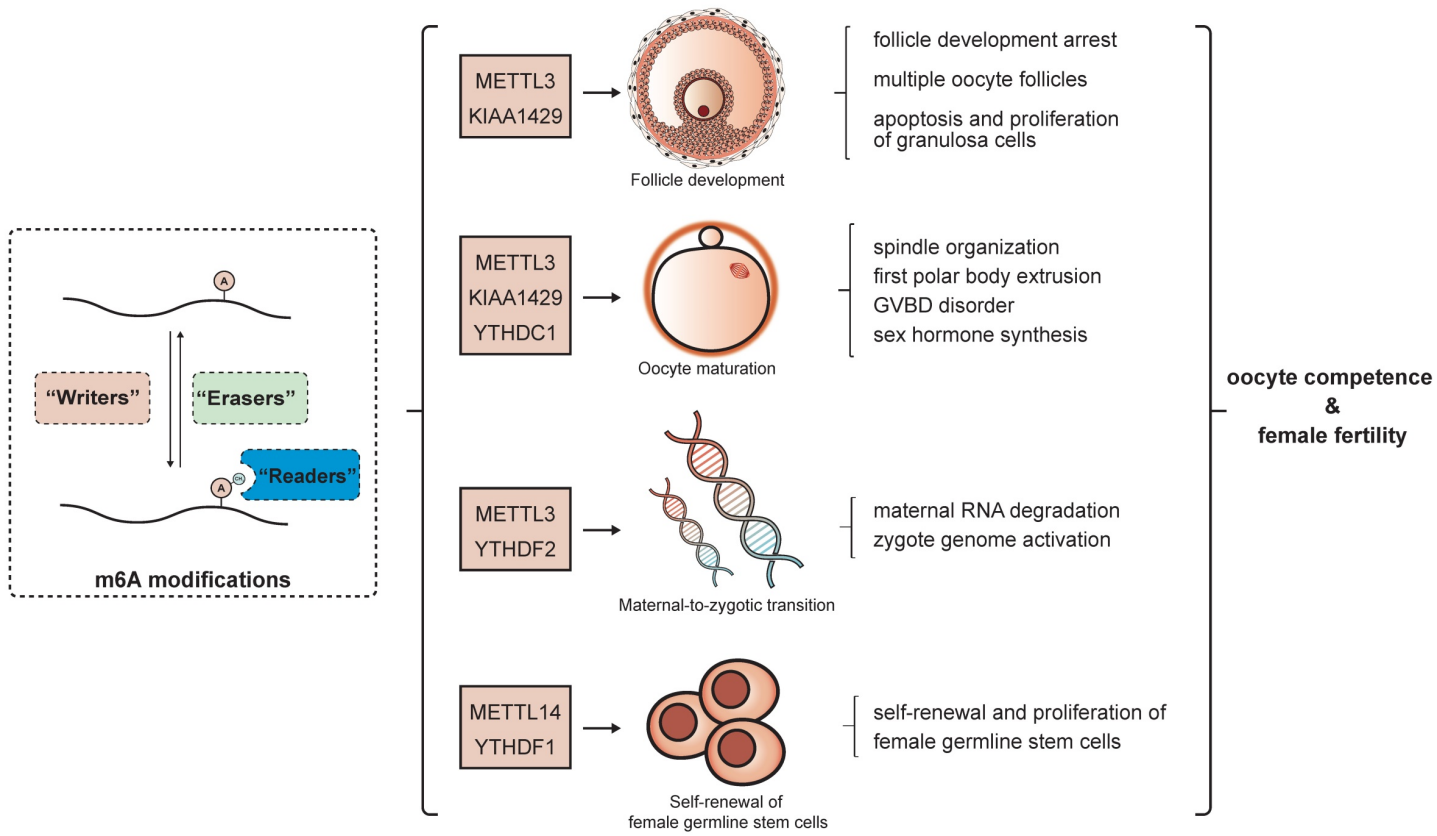
** Role of m^6^A modification in gonad development.** The m^6^A modification regulates oogenesis in terms of follicle development, oocyte maturation, maternal-to-zygote maturation and self-renewal of female germline stem cells, which partially determine oocyte competence and female fertility.

Gene expression is precisely and dynamically controlled during the whole female gonad developmental process. In follicles, proliferation and differentiation of somatic cells relies on the adequate alteration of gene expression patterns, while in oocytes, the changes are even more intricate. Transcription of oocyte genes is active in the early stage of follicle formation and ceases at the GV stage^84^. Maternal mRNAs synthesized in this period dominate the subsequent developmental phases of oocytes and early embryos until zygotic genome activation. As the oocytes emerge from meiotic arrest, maternal mRNAs are constantly degraded and decrease to the lowest level in the two-cell embryo 85. All these processes largely rely on the post-transcriptional regulation of mRNA metabolism and expression. There is wide-ranging evidence for the importance of epigenetic modification in folliculogenesis and oogenesis 31, 86, 87, and the roles of m^6^A modification in these processes have begun to be partially elucidated in recent years. The m^6^A level was found to generally increase along with the development of the mouse ovary, reaching the highest point at the luteal phase. The m^6^A associated regulators also demonstrated a similar trend in accordance with the m^6^A levels, whereby m^6^A writers increased and erasers decreased from 12.5 days post coitum to the mature phase in the ovary, suggesting the potential impact of m^6^A modification during female gonad development 88. There is increasing evidence for the involvement of m^6^A in specific stages and aspects of this process **(Figure 2)**.

### Follicle development

Follicle development is a dynamic and continuous process that happens simultaneously with oocyte growth. Before the resumption of meiosis, oocytes remain in prophase I stage and continue accumulating water, ions, carbohydrates and lipids until GVBD. Follicles provide a developmental microenvironment for oocyte growth and help sustain meiotic arrest 89, 90. MeRIP-seq identified changes in the abundance and distribution of m^6^A modifications in granulosa cells during follicular development 91.

Mettl3 mutation reduced m^6^A levels in zebrafish ovaries. These Mettl3-defective females had smaller gonads than wild-type fish, and a large fraction of follicles failed to fully develop 92. KIAA1429 was found to be expressed in the oocyte nucleus and granulosa cells from follicles in different stages. Increased numbers of primary follicles, decreased secondary, preantral and antral follicles, as well as multiple oocyte follicles were found in *Kiaa1429* ZP3-cKO mice, indicating that *Kiaa1429* deficiency led to arrest and disorganization of follicle developmental. Moreover, *Kiaa1429* deficiency inhibited the proliferation and promoted the apoptosis of granulosa cells. All of these findings suggested that KIAA1429 is required for normal folliculogenesis in mice 93.

### Oocyte maturation

Under the stimulation of LH surge, fully grown oocytes emerge from meiotic arrest and complete subsequent developmental progression up to MII stage prior to ovulation and fertilization. This process, which marks the maturation of oocytes, is largely dependent on precise epigenetic regulation in oocytes 16. The roles of m^6^A in oocyte maturation encompass multiple aspects, including RNA splicing, degradation, transport and translation.

METTL3 expression shares a similar pattern with m^6^A levels in the processes of oogenesis and early embryonic development. They are both abundant in GV and MII oocytes, then dramatically decrease in the zygote and gradually increase during early embryogenesis. *Mettl3* knockdown at the GV stage hindered spindle organization and first polar body extrusion in MII oocytes, which could be partially attributed to decreased translational efficiency of several meiosis-related genes 94. In zebrafish, GVBD disorder and oocyte incompetence were observed in *mettl3*-mutant females. The* mettl3* mutation disturbed sex hormone levels by regulating genes involved in sex hormone synthesis and gonadotropin signaling. A decreased GVBD rate could be partially rescued by hCG or 17α-20β-DHP treatment 92. Oocyte-specific conditional knockout (cKO) of *Kiaa1429* led to a significantly reduced GVBD rate and first polar body extrusion rate. Mechanistically, *Kiaa1429* cKO impaired the correct localization of splicing-related factors SRSF3 and YTHDC1, perturbed the alternative splicing of oogenesis-related transcripts, and led to meiotic arrest 93. The nuclear m^6^A reader YTHDC1 regulates RNA splicing in oocytes by interacting with pre-mRNA processing factors CPSF6, SRSF3, and SRSF7. Loss of YTHDC1 changed the alternative polyadenylation pattern and led to massive alternative splicing defects in the mouse oocyte nucleus 66. It is worth mentioning that both *Kiaa1429*-dificient and *Ythdf1*-dificient oocytes showed abnormal cytoplasmic RNA granules. Considering that both KIAA1429 and YTHDF1 are closely related to m^6^A-mediated RNA splicing, this RNA mislocalization may be caused by inappropriate posttranscriptional processing and transport 66, 93. However, the impact of these RNA granules is still unclear.

### Maternal-to-zygotic transition

The maternal-to-zygotic transition (MZT) consists of selective maternal mRNA degradation and zygotic genome activation. The MZT starts at the resumption of oocyte meiosis rather than fertilization 95. Correct elimination of maternal transcripts lays the foundation for zygotic genome activation and the upcoming embryogenesis 16, 96, and is part of the oocyte competence built up in preceding developmental processes. It has been demonstrated that MZT is partially controlled m^6^A modification.

*Mettl3* knockdown impeded embryonic development from the two-cell stage to four-cell stage in activated and diploidized MII oocytes. The *Mettl3*-mropholino treated group showed remarkably decreased global transcription. RNA-seq assays detected hundreds of upregulated genes, part of which are enriched in GO terms related to RNA metabolic processes and cell cycle, demonstrating that *METTL3* knockdown may affect MZT by impairing mRNA degradation^94^. The clearance of maternal RNAs was disturbed in *ythdf2*-dificient zebrafish. Upregulated maternal transcripts and downregulated zygotic transcripts were observed in both *ythdf2* homozygous mutant oocytes and *ythdf2*-morpholino treated oocytes. The *ythdf2*-dificiency-meditated RNA retention occurred in a m^6^A preferential manner, leading to obvious embryonic developmental delay in zebrafish 97. In mice, *Ythdf2* knockout did not affect follicle development or oocyte meiosis, but caused serious embryonic developmental defects, resulting in complete female infertility. YTHDF2 deficiency mainly affected gene expression in MII oocytes instead of GV oocytes. This suggested that YTHDF2 did not regulate the maternal transcriptome but was required to maintain the appropriate transcript dosage 98.

### Self-renewal of female germline stem cells

The discovery of female germline stem cells (FGSCs) provided a theoretical basis to obtain additional oocytes even after birth. Zou et al. established a neonatal mouse FGSC line and produced offspring using FGSCs retrieved from adult mice, providing a novel idea for research on oogenesis 99.

RNA m^6^A profiling revealed differential m^6^A patterns between FGSCs and somatic STO cells. Compared to STO cells, FGSCs had higher m^6^A abundance and manifested dissimilar mRNA expression levels of m^6^A regulators. Knockdown of the m^6^A reader *YTHDF1* hindered the proliferation of FGSCs, but the specific mechanisms have not yet been explored 100. METTL14 participates in the self-renewal of FGSCs by facilitating the m^6^A modification of circGFRα1, which acts as a sponge of miR-449 and promotes the expression of their common target GFRα1 to sustain FGSCs proliferation. *METTL14* knockdown resulted in decreased m^6^A modification of circGFRα1, which did not change its expression level but led to its nuclear retention. Similarly, m^6^A-motif-mutated circGFRα1 mainly accumulated in the nucleus after its overexpression, suggesting that cytoplasmic transport was dependent on m^6^A 101. All these findings support the involvement of m^6^A in postnatal oocyte production from FGSCs.

## m^6^A in female reproductive diseases

There is evidence that m^6^A modification takes part in the process of inflammation and immune activity 102, 103. Considering that inflammatory or immune disorders play a critical role in many female reproductive diseases such as endometriosis and adenomyosis, polycystic ovary syndrome, preeclampsia and spontaneous abortion 104, 105, it stands to reason that m^6^A modification may also contribute to the pathological mechanisms underlying these diseases.

### Endometriosis and adenomyosis

Bioinformatic analyses revealed that most of the m^6^A regulators in the eutopic endometrium were aberrantly expressed compared to normal or eutopic endometrium. Among all the regulators, *HNRNPA2B1* and *HNRNPC* were negatively correlated with the severity of endometriosis, and might influence infiltrating immune cells 106. The m^6^A content was found to be dramatically decreased in eutopic and ectopic samples, which was consistent with decreased METTL3 and increased ALKBH5 levels. METTL3 regulates the migration and invasion of endometrial stromal cells by facilitating DGCR8-mediated maturation of miR126, contributing to endometriosis development 107. Similarly, the expression pattern of m^6^A regulators in the myometrium was also found to be different from normal tissues. The cluster of m^6^A regulators may potentially affect the immune response and cell adhesion according to data mining, playing roles in the myometrium dysfunction 108.

### Polycystic ovary syndrome

Polycystic ovary syndrome (PCOS) is often accompanied by insulin resistance. FTO overexpression diminished m^6^A modification in *FLOT2* transcripts and enhanced its expression in granulosa cell line KGN. FLOT2 is associated with insulin resistance and was found to be upregulated in granulosa cells from PCOS patients. FTO was found to regulate cell proliferation, apoptosis and insulin resistance in KGN cells through a FLOT2-dependent manner, indicating that FTO might participate in regulating insulin resistance in PCOS 109. Luteinized granulosa cells from non-obese PCOS patients had higher m^6^A levels and exhibited a dissimilar distribution pattern of m^6^A peaks in comparison to the control group. Mechanistically, the *FOXO3* transcript was m^6^A modified and underwent YTHDF2‐mediated decay in controls, which was in contrast to the samples from PCOS patients. Reduced m^6^A modification of the *FOXO3* mRNA in controls enhanced the expression of FOXO3 after *METTL3* or *METTL14* knockdown, while having the inverse effect after *FTO* knockdown. However, this effect was absent from the PCOS granulosa cells^110^. These findings illustrate a potential mechanism driving insulin resistance in PCOS patients.

### Premature ovarian insufficiency

In addition to increased m^6^A modification in naturally aging mouse ovaries 111, the levels of m^6^A modification ware also found to be obviously upregulated in either ovarian tissues or human granulosa cells from premature ovarian insufficiency (POI) patients. This elevation was associated with a relative reduction of FTO compared to ALKBH5. *FTO* knockdown impeded cell proliferation, alleviated apoptosis and disturbed marker expression in human granulosa cells^112^. Furthermore, m^6^A was also increased in a mouse model of cyclophosphamide (CTX)-induced premature ovarian insufficiency in a concentration- and time-dependent manner. CTX treatment upregulated the m^6^A writers METTL3, METTL14, ZC3H13 and KIAA1429, while downregulating the demethylase FTO and several m^6^A readers^112^, ^113^.These results indicate that m^6^A modification may act as a potential biomarker of ovarian dysfunction 112, 113.

### Preeclampsia

Dysfunction of the placenta in preeclampsia and eclampsia patients usually leads to abnormal fetal growth and serious complications for the pregnant women. Previous studies have identified the role of epigenetics in preeclampsia and fetal growth restriction 114, 115. The potential involvement of m^6^A modifications in the pathological processes of preeclampsia has been proposed based on several lines of evidence. MeRIP-seq analysis demonstrated a correlation between higher m^6^A levels at the 5′‐UTR in placental mRNAs and small‐for‐date children, whereas decreased m^6^A levels near stop codons were dominant in heavy‐for‐date placenta samples. Higher SMPD1 protein levels in preeclampsia placenta samples, in particular, are more likely to result from m^6^A enrichment at 5′‐UTR instead of increased mRNA levels 116. Enhanced METTL3 expression was found to be responsible for increased m^6^A methylation in placental trophoblasts from preeclampsia patients. These aberrant m^6^A changes lead to the upregulation of hnRNPC1/C2 expression, which may induce vitamin D deficiency by inducing VDR and trophoblast dysfunction 117. Furthermore, METTL3 was found to facilitate the maturation of miR-497-5p/195-5p by mediating DGCR8's recognition of pri-miR-497-5p/195-5p in an m^6^A-dependent manner. Increased miR-497-5p/195-5p in the preeclampsia-affected placenta was found to suppress WWP1 expression, disturbing trophoblast proliferation, migration, and invasion, eventually aggravating preeclampsia progression 118.

### Recurrent miscarriage/spontaneous abortion

The pathogenesis of recurrent miscarriage (RM) has not been fully elucidated yet. In addition to the known factors including genetic, endocrine, anatomical, and immunological disorders 119, epigenetic abnormalities are also part of the underlying mechanisms 120. So far, the research on the role of m^6^A in RM has mainly focused on its effects on trophoblast function. Higher ALKBH5 expression and lower m^6^A levels were found in chorionic villi from RM patients. ALKBH5 diminished m^6^A modification of *CYR61* mRNA, reducing its stability and subsequent protein expression. These changes inhibited the proliferation and invasion of trophoblast cell at the maternal-fetal interface in early pregnancy, suggesting that ALKBH5 may be a potential key factor in the pathogenesis of RM 121. The m^6^A modification was also found to be involved in BPDE-related RM. BPDE, a metabolite of environmental benzo(a)pyrene, was found to upregulate lncHZ01 in trophoblast cells, and lncHZ01-meditated MXD1 upregulation promoted *METTL14* transcription, which increased m^6^A modification and enhanced the stability of lncHZ01, forming a positive feedback loop that eventually inhibited trophoblast cell proliferation and induced miscarriage^122^. Higher m^6^A levels and several aberrantly expressed m^6^A regulators were found in spontaneous abortion (SA) patients. FTO, in particular, was significantly downregulated in the chorionic villi and trophoblasts of SA patients. Lower FTO changed the m^6^A patterns of several genes involved in immunotolerance, immune cell infiltration and angiogenesis, demonstrating a possible role of m^6^A in SA progression 123.

## Conclusions

The research on m^6^A RNA modification has expanded broad horizons for researchers and uncovered several new epigenetic mechanisms underlying a number of pathophysiological processes. This reversible modification is controlled by three kinds of regulators and influences various RNA-related processes such as RNA degradation, splicing, transport and translation, regulating gene expression from multiple aspects at the post-transcriptional level. The involvement of m^6^A modifications has been observed in various bioprocesses including cell differentiation, development, metabolism, stress response and other cellular activities 2, 13-15, serving as an important epigenetic regulatory mechanism underlying both physiological and pathological processes.

In the female reproductive system, m^6^A modifications regulate multiple stages of oogenesis, including follicle development, oocyte maturation, and the maternal-to-zygotic transition, as well as the self-renewal of female germline stem cells artificially established *in vitro*. Furthermore, m^6^A participates in the pathogenesis of a variety of female reproductive diseases including endometriosis and adenomyosis, polycystic ovary syndrome, premature ovarian insufficiency, preeclampsia, and recurrent miscarriage, all of which underscore the impact of m^6^A on female fertility.

Recently several researches have revealed the clinical potential of m^6^A-targeting strategies. METTL3 inhibitors exhibited the promising anti-cancer effect against acute myeloid leukemia 124, 125. pharmacological inhibition of FTO significantly suppressed leukemia stem/initiating cell self-renewal and sensitized leukemia cells to T cell cytotoxicity^126^. However, m^6^A was only considered as a prospective therapeutic target of cancer treatment in current evidences 127. Considering its crucial participation in female gonad development and reproductive diseases, m^6^A-targeting interventions should also be taken into account as promising approaches in treatment of infertility and reproductive disorders.

Nevertheless, our understanding of the full impact of m^6^A is far from complete. For example, how different m^6^A readers recognize m^6^A-modified transcripts and lead to different transcript outcomes is still unclear. More importantly, translational studies aiming to utilize m^6^A as a novel target for therapy are in their infancy. All these unsolved questions merit further studies in the future.

## Figures and Tables

**Table 1:** Role of m^6^A in female reproductive diseases.

diseases	m^6^A regulator	aberrant expression	target	effect of m^6^A on target RNA	effect of m^6^A in diseases	reference
Endometriosis	METTL3	↓	miR-126	Enhanced DGCR8-mediated maturation of pri-miR126	Decreased METTL3 and m^6^A promoted the migration and invasion of endometrial stromal cells in endometriosis	107
Polycystic ovary syndrome	FTO	-	FLOT2	FTO overexpression diminished m^6^A modification in FLOT2 transcripts and enhanced its expression in granulosa cell line KGN	FTO might regulate insulin resistance process in PCOS by enhancing FLOT2 expression	109
	YTHDF2	-	FOXO3	Lower m^6^A levels inhibited YTHDF2-meditated FOXO3 transcript decay	m^6^A might regulate function of granulosa cells by controlling FOXO3 expression	110
Premature ovarian insufficiency	FTO	↓	-	-	FTO knockdown impeded cell proliferation, alleviated cell apoptosis and disturbed expression of cell markers in human granulosa cells	112
Preeclampsia	-	-	SMPD1	m^6^A modification of SMPD1 at the 5′‐UTR promoted protein translation at posttranscriptional level	m^6^A levels in placenta were related to birth weight	116
	METTL3	↑	hnRNPC1/C2	METTL3 knockdown significantly reduced hnRNPC1/C2 expression in trophoblast cells	-	117
	METTL3	-	miR-497-5p/195-5p	METTL3 facilitated maturation of miR-497-5p/195-5p by mediating recognition of pri-miR-497-5p/195-5p by DGCR8	METTL3-meditated upregulation of miR-497-5p/195-5p disturbed trophoblast proliferation, migration, and invasion, eventually aggravating preeclampsia progression	118
Recurrent miscarriage/ spontaneous abortion	METTL14	↑	lncHZ01	METTL14 stabilized lncHZ01 and formed a positive feedback loop with LncHZ01	METTL14 enhanced lncHZ01 expression to inhibit trophoblast cell proliferation and induces miscarriage.	122
	ALKBH5	↑	CYR61	ALKBH5 diminished m^6^A modification on CYR61 mRNA, impaired its stability and subsequent protein expression	inhibited proliferation and invasion of trophoblast at the maternal-fetal face in early pregnancy	121
